# Metrnl as a predictive biomarker for postprandial hypertriglyceridemia in overweight and obese populations

**DOI:** 10.3389/fendo.2026.1729571

**Published:** 2026-02-12

**Authors:** Xiaoyu Wang, Yale Tang, Shaojing Zeng, Luxuan Li, Yilin Hou, Dandan Liu, Peipei Tian, Guangyao Song

**Affiliations:** 1 Department of Internal Medicine, Hebei Medical University, Shijiazhuang, Hebei, China; 2 Department of Endocrinology, Hebei General Hospital, Shijiazhuang, Hebei, China; 3 Department of International Medical, Shijiazhuang People’s Hospital, Shijiazhuang, Hebei, China

**Keywords:** adipokine meteorin-like protein, Metrnl, oral fat tolerance test, overweight and obesity, postprandial hypertriglyceridemia

## Abstract

**Purpose:**

The relationship between adipokine meteorin-like protein (Metrnl) and postprandial hypertriglyceridemia (PHTG) in overweight and obese populations remains unclear. This study examined the association between serum Metrnl and PHTG with normal fasting lipid profiles, using a standardized oral fat tolerance test (OFTT) to classify fat tolerance. The aim was to explore potential therapeutic targets for early obesity intervention.

**Patients and methods:**

We enrolled 105 adults with normal fasting lipid profiles who met Chinese lipid management criteria for low-risk atherosclerotic cardiovascular disease (ASCVD) prevention. Participants were grouped as control (CON), overweight (OW), or obese (OB). All underwent an OFTT, with venous blood collected fasting serum Metrnl, total cholesterol (TC), triglycerides (TG), high-density lipoprotein cholesterol (HDL-C), low-density lipoprotein cholesterol (LDL-C), fasting insulin (FINS). Venous blood samples were collected at 1, 2, 3, and 4 hours postprandially to quantitatively analyze the dynamic changes in serum lipid profiles.

**Results:**

Serum Metrnl showed a significant negative correlation with PHTG (r = –0.473, P < 0.001), fasting TG (r = –0.370, P < 0.001), FINS (r = –0.261, P = 0.007). Multivariate regression identified fasting TG as a risk factor for PHTG. Each 0.1 mmol/L increment in fasting triglycerides was significantly associated with a 76.9% higher risk of PHTG. Metrnl was identified as protective (OR = 0.211, P < 0.001), the protective cutoff for Metrnl was 2.11ng/ml. A combined model of fasting TG and Metrnl improved PHTG prediction over fasting TG or Metrnl alone, with ROC analysis showing an AUC of 0.908, sensitivity of 82.7%, and specificity of 90.6%.

**Conclusions:**

Overweight and obese adults with normal fasting lipid profiles are at high risk of PHTG. Low serum Metrnl is closely associated with early lipid abnormalities and insulin resistance. Combining Metrnl with TG enhances diagnostic accuracy for PHTG.

## 1 Introduction

Overweight and obesity are escalating global public health issues. By 2030, it is projected that more than 2.9 billion adults worldwide will have a high body mass index (BMI ≥ 25 kg/m^2^), with 1.1 billion meeting the criteria for obesity (1). Overweight and obesity result from excessive accumulation or abnormal distribution of adipose tissue, particularly triglycerides, and are often accompanied by dysregulated adipokine secretion (2). This dysregulation impairs the regulation of appetite, satiety, fat distribution, and insulin secretion (3). Obesity and hyperlipidemia can promote insulin resistance, which in turn increases the risk of vascular diseases (4). Meteorin-like protein (Metrnl) is a novel adipokine primarily expressed in white adipose tissue and widely distributed across human tissues. It regulates blood triglycerides (TG) levels, exhibits anti-atherosclerotic effects, and improves insulin resistance (5, 6). Clinical trials and studies in Metrnl-deficient mice have demonstrated its beneficial role in lipid metabolism. However, findings regarding circulating Metrnl levels in obese patients remain inconsistent (7–14), likely due to the influence of multiple factors.

This study employed a standardized oral fat tolerance test (OFTT) in overweight and obese individuals with normal fasting lipid profiles to observe postprandial changes in lipid profiles and insulin secretion, investigate the relationship between overweight/obesity and postprandial hypertriglyceridemia (PHTG), and explore the correlation between Metrnl and these conditions. The findings aim to provide new insights for atherosclerotic cardiovascular disease (ASCVD) risk assessment and identify potential therapeutic targets for obesity-related metabolic disorders.

## 2 Materials and methods

### 2.1 Study participants

This study complied with the Declaration of Helsinki and was approved by the Hebei Provincial Ethics Committee. It was registered with the Chinese Clinical Trial Registry (Registration Number: ChiCTR2100048497). In 2024, participants were recruited from outpatient clinics. Eligible participants were aged 25 to 69 years and classified as low risk for primary prevention of ASCVD, as defined by the Chinese Lipid Management Guidelines (15), with normal fasting lipid levels (total cholesterol (TC) < 5.2 mmol/L, low-density lipoprotein cholesterol (LDL-C) < 3.4 mmol/L, triglycerides (TG) < 1.7 mmol/L, high-density lipoprotein cholesterol (HDL-C) < 4.1 mmol/L) and without diabetes.

### 2.2 Exclusion criteria

1. Individuals with a family history of endocrine-related diseases or secondary dyslipidemia due to hypothyroidism, Cushing’s syndrome, immune disorders, cancer, or excessive alcohol consumption.
2. Use of lipid-lowering drugs, fish oil, thiazides, non-selective beta-blockers, glucocorticoids, or contraceptives within the past three months.
3. History of severe infections, surgery, trauma, or psychiatric disorders.
4. Food or drug allergies, or intolerance to high-fat or high-protein foods.
5. Inability to undergo multiple venipunctures due to needle or blood phobia, as assessed by questionnaire.

### 2.3 Standardized OFTT

Participants followed a standard diet (avoiding high-fat and high-protein foods) for one week before the test. After fasting from 10:00 PM the previous day, participants consumed a standardized OFTT meal at 8:00 AM the following morning (16). The high-fat meal contained 700 kcal, with 60% from fat (46.7 g), 25% from protein (43.8 g), and 15% from carbohydrates (26.3 g). The fat composition included 14.5 g saturated fatty acids, 12.1 g medium-chain triglycerides, 21.4 g monounsaturated fatty acids, and 10.9 g polyunsaturated fatty acids. Participants consumed the meal within 10 minutes and refrained from eating or drinking (except water) for 4 hours. Smoking and vigorous exercise were prohibited. Venous blood samples were collected at 0, 1, 2, 3, and 4 hours postprandially, centrifuged immediately, and stored at –80°C.

### 2.4 Biochemical measurements

Fasting blood glucose (FBG), TC, TG, HDL-C, and LDL-C serum creatinine (Scr) were measured using an automated biochemical analyzer (Hitachi, Japan). Fasting insulin (FINS) was quantified by electrochemiluminescence. Metrnl concentrations were determined using an ELISA kit (FineTest). cystatin C (CysC) and β2-microglobulin(β2-MG) were measured using ELISA kits (Jiangsu Aidisheng Biotechnology). BMI was calculated as weight divided by height squared (kg/m^2^). The triglyceride-glucose index (TyG) was computed as LN [fasting TG (mg/dL) × fasting BG (mg/dL)/2]. The homeostasis model assessment of insulin resistance (HOMA-IR) was calculated as [FBG (mmol/L) × FINS (μIU/mL)]/22.5. The endogenous creatinine clearance rate (eCCr) was estimated using the Cockcroft-Gault formula: [(140 – age) × weight (kg)]/[0.818 × Scr (μmol/L)]; for females, the result was multiplied by 0.85.

### 2.5 Group classification

According to the 2024 Chinese guidelines for obesity diagnosis (17), participants were categorized into normal weight (CON, BMI 18.5-23.9 kg/m^2^), overweight (OW, BMI 24.0-27.9 kg/m^2^), and obese (OB, BMI ≥ 28.0 kg/m^2^) groups. Based on the 2016 European lipid consensus and a 2019 study on postprandial triglycerides (PTG) in overweight populations, PHTG was defined as PTG ≥2.0 mmol/L at any postprandial time point (18). Participants were classified into non-PHTG (NPHTG, PTG < 2.0 mmol/L) and PHTG (PTG ≥ 2.0 mmol/L) groups after the OFTT.

### 2.6 Statistical analysis

Data were analyzed using SPSS version 27.0 and GraphPad Prism version 9.0. The Shapiro–Wilk test assessed the normality of continuous variables. Normally distributed data are presented as mean ± standard deviation (X ± s), whereas non-normally distributed data are expressed as median (Q1, Q3). Group comparisons were conducted using one-way ANOVA, the Kruskal–Wallis test, or the Bonferroni *post hoc* test, as appropriate. Categorical variables are reported as counts and percentages (n, %), with comparisons performed using chi-square tests. Pearson or Spearman correlation analysis was applied depending on data distribution and variance homogeneity. Univariate and multivariate logistic regression analyses were conducted to evaluate the relationship between serum Metrnl and PHTG, and results are reported as odds ratios with 95% confidence intervals. Receiver operating characteristic (ROC) curve analysis was used to compare the predictive performance of single indicators and combined models for PHTG, with DeLong’s test applied to assess differences in AUC values. Statistical significance was set at P < 0.05 (two-tailed).

## 3 Results

### 3.1 Comparison of adipokine Metrnl and baseline data among BMI-based groups

The study included 105 participants: 34 in the CON group, 39 in the OW group, and 32 in the OB group. All groups completed the OFTT with good tolerance. No significant differences were observed in sex or age among the groups. Among the three groups, blood pressuren levels, FBG, LDL-C, Scr, eCcr, β2-MG, CysC gradually increased (P < 0.05). Moreover, waist-to-hip ratio (WHR),waist-to-height ratio (WHtR), FINS, HOMA-IR, TG, and TyG were significantly higher in both the OW and OB groups compared to the CON group (P < 0.001). The OB group exhibited even higher WHtR and diastolic blood pressure (DBP)levels than the OW group (P < 0.05), whereas serum Metrnl levels and HDL-C levels were lower in the OW and OB groups compared with the CON group, OB group significantly lower (P < 0.05) (**Table 1**). These data indicate that individuals who are overweight or obese, despite having normal fasting blood lipid profiles, may already show early dyslipidemia, insulin resistance, and kidney dysfunction associated with obesity, as well as decreased circulating levels of the adipokine Metrnl.

**Table 1:** Comparison of baseline data among BMI groups.

Variable	CON (n = 34)	OW (n = 39)	OB (n = 32)	*Pvalue*
Age (Years)	44 (33, 54)	53 (39, 58)	45 (39, 56)	0.101
Sex, male (n, %)	9 (26.5)	18 (46.2)	15 (46.9)	0.149
BMI (kg/m^2^)	22.09 (21.05,23.26)	25.69 (25.07, 26.66)^**^	29.01 (28.20, 31.17)^**#^	<0.001
WHR	0.81 ± 0.06	0.86 ± 0.05^**^	0.89 ± 0.06^**^	<0.001
WHtR	0.46 ± 0.04	0.52 ± 0.03^**^	0.57 ± 0.05^**##^	<0.001
SBP (mmHg)	117.88 ± 10.55	123.56 ± 11.87	124.84 ± 14.52^*^	0.052
DBP (mmHg)	76.41 ± 7.05	77.10 ± 6.79	83.19 ± 8.96^**#^	<0.001
FBG (mmol/L)	5.09 (4.83, 5.57)	5.28 (5.02, 5.74)	5.48 (5.17, 5.77)^*^	0.023
FINS (µIU/mL)	6.17 (4.25, 8.67)	10.44 (8.17, 12.84)^**^	11.61 (7.77, 14.32)^**^	<0.001
HOMA-IR	1.37 (0.91, 2.15)	2.47 (1.86, 3.31)^**^	2.96 (1.92,3.73)^**^	<0.001
TC (mmol/L)	4.23± 0.71	4.30± 0.48	4.35± 0.52	0.755
TG (mmol/L)	0.69 (0.59, 0.93)	1.05 (0.86, 1.32)^*^	1.16 (0.85, 1.42)^**^	<0.001
HDL-C (mmol/L)	1.41± 0.29	1.22± 0.19^**^	1.20± 0.24^**^	<0.001
LDL-C (mmol/L)	2.53± 0.55	2.80 ± 0.49^*^	2.85 ± 0.51^*^	0.025
TyG index	8.04 ± 0.35	8.41 ± 0.37^**^	8.46 ± 0.33^**^	<0.001
Scr (µmol/L)	63.47 ± 12.76	68.56 ± 10.93	74.80 ± 14.70^**^	0.002
eCCr (mL/min)	100.78 ± 20.43	111.94 ± 25.19	116.59 ± 28.05^*^	0.030
β2-MG (µg/L)	34.48 ± 10.43	43.62 ± 15.30^*^	48.85 ± 21.98^*^	0.001
CysC (ng/mL)	33.29 (25.29,42.68)	40.60 (30.06, 60.99)	48.61 (31.05, 64.49)^*^	0.008
Metrnl (ng/mL)	2.33 (2.10,3.25)	2.23 (1.98,2.84)	2.05 (1.53,2.62)^*^	<0.001

Means ± SD for normally distributed variables or medians (interquartile range) for non–normally distributed variables.^*^P < 0.05 vs. CON group; ^**^P < 0.001 vs. CON group. *^#^P* < 0.05 vs. OW group; *^##^P* < 0.001 vs. OW group.

CON, control group; OW, overweight group; OB, obese group; BMI, body mass index; WHR, waist-to-hip ratio; WhtR, waist-to-height ratio;SBP, systolic blood pressure;DBP, diastolic blood pressure; FBG, fasting blood glucose; FINS, fasting insulin; HOMA-IR, homeostasis model assessment of insulin resistance; TC, total cholesterol; TG, triglyceride; HDL-C, high-density lipoprotein-cholesterol; LDL-C, low-density lipoprotein-cholesterol;TyG index, triglyceride–glucose index; Scr, serum creatinine; eCCr, endogenous creatinine clearance rate; β2-MG,β2-microglobulin; CysC, cystatinC; Metrnl, meteorin-like protein.

### 3.2 Comparison of OFTT data across BMI-stratified groups at different times and incidence of PHTG

**Figure 1:**
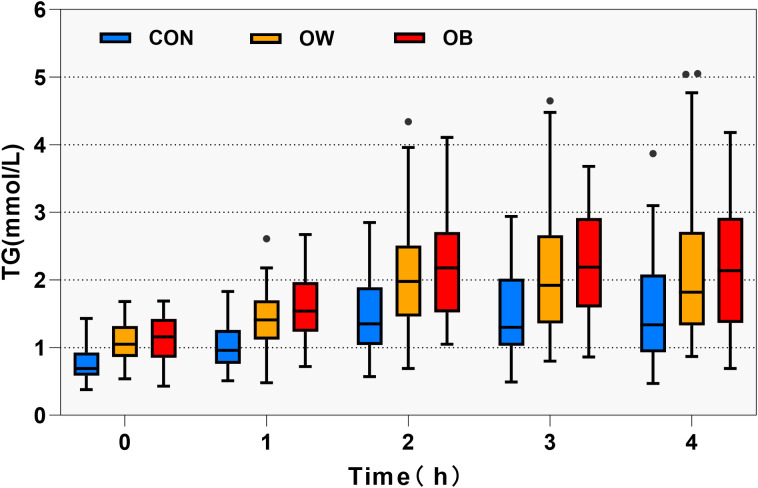
Trend of TG levels at different time points during the OFTT. The box plot illustrates the distribution of triglyceride (TG) levels in three subject groups at different time points during the Oral Fat Tolerance Test (OFTT). Boxes represent interquartile ranges (IQRs, 25th–75th percentiles), with horizontal lines indicating medians; whiskers extend to 1.5×IQR (non-outlier range), and outliers are plotted as individual points.

**Figure 2:**
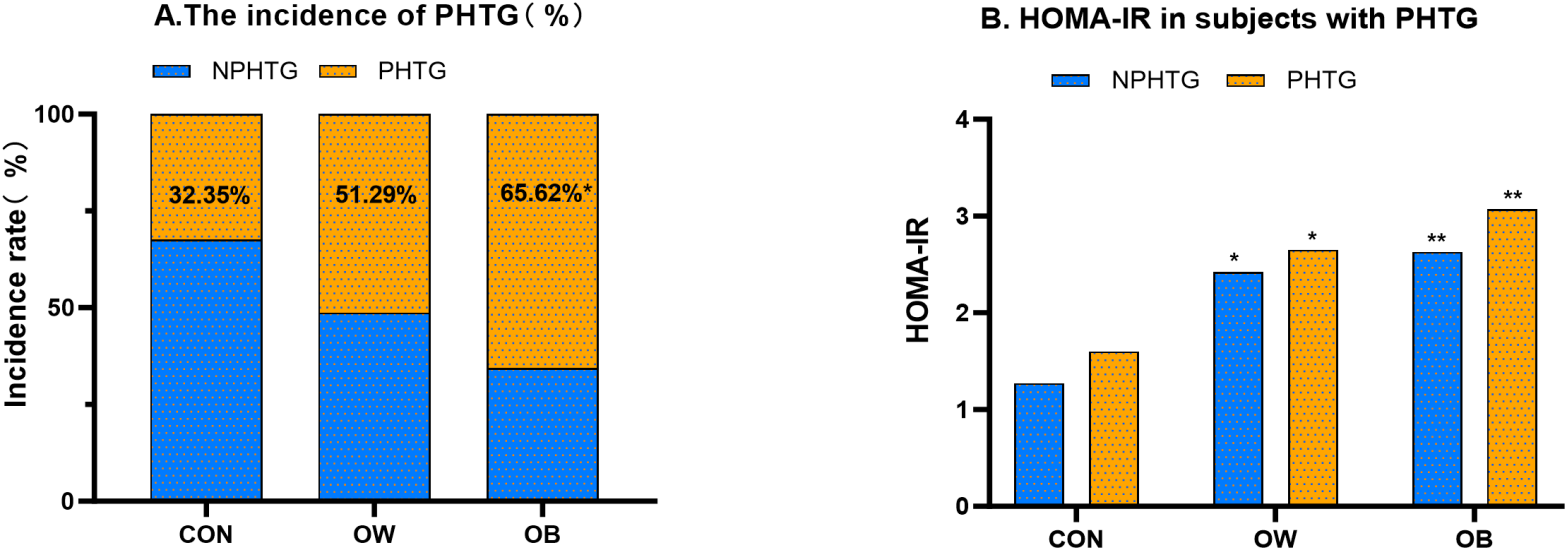
Incidence of PHTG and HOMA-IR levels in subjects across different BMI groups. **(A)** Incidence of postprandial hypertriglyceridemia (PHTG) and non-hypertriglyceridemia (NPHTG) in different body weight groups. CON: Normal weight group; OW: Overweight group; OB: Obese group. ^*^P < 0.05 vs. CON group; ^**^P < 0.001 vs. CON group. **(B)** Comparison of HOMA-IR between subjects with postprandial hypertriglyceridemia (PHTG) and non-postprandial hypertriglyceridemia (NPHTG) across different BMI groups. ^*^P < 0.05 vs. CON group; ^**^P < 0.001 vs. CON group.

During the 0-4hour OGTT period, no statistically significant differences were observed in TC levels among the three groups at any time point (P > 0.05).TG and LDL-C levels were significantly elevated in the OW and OB groups relative to the CON group, especially the TG level (P < 0.001) (**Table 2**, **Figure 1**). HDL-C concentrations were significantly lower in the OW and OB groups than in the CON group (P < 0.001) (**Table 2**). Based on TG measurements at each time point during the OFTT and the diagnostic criteria for PHTG (18), the incidence of PHTG was 32.35% in the CON group, while the OW and OB groups exhibited significantly higher rates of 51.29% and 65.62%, respectively. A significant difference in PHTG incidence was observed between the OW and CON groups (P < 0.05) (**Figure 2A**). As an indicator of insulin resistance, the HOMA-IR values in CON group maintained the normal range, whereas both the OW and OB groups showed markedly elevated levels compared to the CON group (P < 0.05). After stratifying the three groups into PHTG and NPHTG subgroups, HOMA-IR values were consistently higher in the PHTG subgroup than in the non-PHTG subgroup. Notably, OB individuals with PHTG exhibited significantly elevated HOMA-IR levels compared to those in the CON group (P < 0.05) (**Figure 2B**). The findings indicate that individuals with overweight or obesity are more susceptible to PHTG and exhibit persistently lower HDL-C levers following meals. Moreover, obese individuals with PHTG demonstrate heightened insulin resistance.

### 3.3 Correlation analysis between Metrnl and clinical indicators

Metrnl levels showed no significant correlation with age or gender, indicating that its expression is independent of these demographic factors. Metrnl demonstrated inverse associations with various indicators of glycolipid metabolism, including BMI, WHR, WHtR, TG, TyG index, FINS, HOMA-IR, and the prevalence of PHTG, with the strongest negative correlation observed for PHTG (r = –0.473, P < 0.001). Regarding obesity-related renal impairment, Metrnl showed a positive correlation with eCCr (r = 0.238, P = 0.015) and significant negative correlations with Scr, β2-MG, and CysC (**Table 3**). These findings further substantiate the role of the adipokine Metrnl in glycolipid metabolism among overweight and obese populations and, for the first time, reveal a significant inverse association between circulating Metrnl levels and early-stage obesity-related renal injury.

### 3.4 Binary logistic regression analysis of Metrnl and PHTG

**Figure 3:**
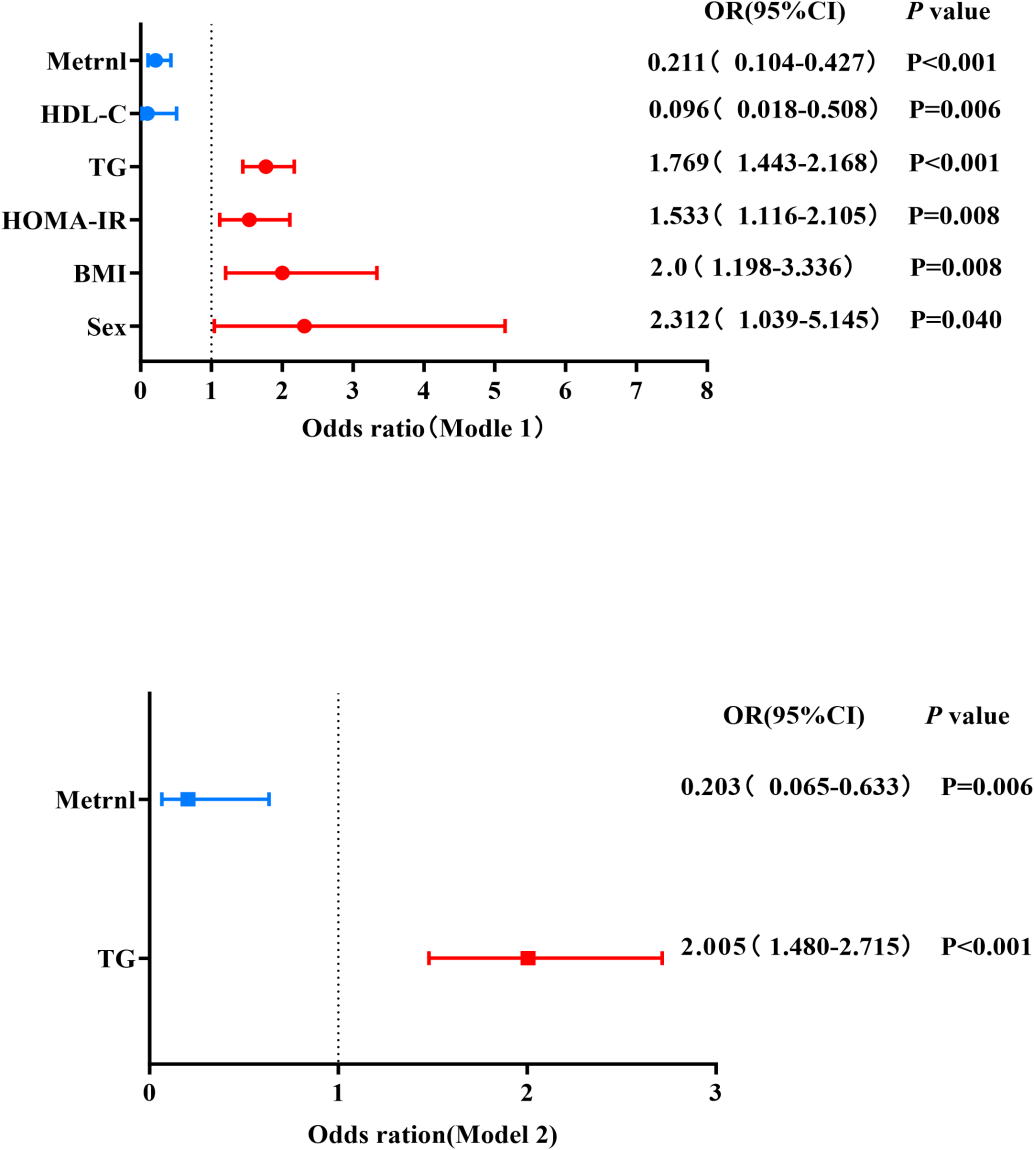
Binary logistic regression analysis of influencing factors for PHTG. Model 1: Univariate binary logistic regression analysis; Model 2: Multivariate binary logistic regression analysis after adjusting for sex, age, BMI, HOMA-IR, eCCr and HDL-C. The x-axis represents the odds ratio (OR), and the horizontal lines represent the 95% confidence interval (95%CI). OR < 1 indicates a protective factor for PHTG, while OR > 1 indicates a risk factor for PHTG.

To further investigate the association between Metrnl and PHTG, a univariate logistic regression analysis (Model 1) was performed with PHTG as the dependent variable and indicators that showed statistically significant differences in **Table 2** as independent variables. The results indicated that male sex (OR = 2.312, P = 0.040), BMI (OR = 2.0, P = 0.008), HOMA-IR(OR = 1.533, P = 0.008), TG×10 (OR = 1.769, P < 0.001) were risk factors for PHTG, whereas Metrnl (OR = 0.211, P < 0.001) and HDL-C (OR = 0.096, P = 0.006) were identified as protective factors (**Table 4**, **Figure 3**). After adjusting age and sex for confounding variables, a multivariate logistic regression analysis (Model 2) incorporating the above indicators as independent variables revealed that fasting TG×10 (OR = 2.005 P < 0.001) remained a risk factor for PHTG, while Metrnl (OR = 0.203, P = 0.006) was confirmed as a protective factor (**Table 4**, **Figure 3**). To enhance the statistical model and improve the accuracy of data interpretation, triglyceride concentrations were rescaled by a factor of ten. The reported estimates represent the change in the probability of outcome events per 0.1 mmol/L increase in fasting triglyceride levels. The results indicate that each 0.1 mmol/L increment in fasting triglycerides was significantly associated with a 76.9% higher risk of PHTG.

**Table 2:** Plasma lipids, blood glucose, and insulin concentrations in BMI groups during OFTT at different time points.

Time	Before 0h	After 1h	After 2h	After 3h	After 4h
TC (mmol/L)					
CON	4.39 (3.82,4.81)	4.53 (3.83,4.87)	4.45 (3.80,4.78)	4.41 (3.79,4.79)	4.15 (3.82,4.62)
OW	4.32 (4.09,4.64)	4.28 (4.05,4.61)	4.29 (3.96,4.82)	4.29 (3.96,4.82)	4.46 (3.98,4.91)
OB	4.39 (3.98,4.76)	4.34 (4.03,4.67)	4.57 (4.05,4.76)	4.49 (4.16,4.76)	4.67 (3.92,5.09)
*P*value	0.974	0.820	0.773	0.418	0.095
TG (mmol/L)					
CON	0.69 (0.59,0.93)	0.96 (0.76,1.26)	1.35 (1.04,1.90)	1.30 (1.03,2.02)	1.34 (0.93,2.08)
OW	1.05 (0.86,1.32)^**^	1.41 (1.12,1.70)^**^	1.98 (1.46,2.51)^**^	1.92 (1.36,2.66)^*^	1.82 (1.33,2.71)^*^
OB	1.18 (0.85,1.43)^**^	1.53 (1.22,2.03)^**^	2.17 (1.50,2.71)^**^	2.12 (1.58,2.84)^**^	2.18 (1.39,2.94)^*^
Pvalue	<0.001	<0.001	<0.001	<0.01	0.003
HDL-C (mmol/L)					
CON	1.46 (1.19,1.63)	1.44 (1.20,1.63)	1.40 (1.19,1.55)	1.40 (1.20,1.59)	1.38 (1.19,1.53)
OW	1.18 (1.07,1.37)^**^	1.21 (1.06,1.36)	1.23 (1.07,1.35)^**^	1.17 (1.02,1.28)^**^	1.15 (1.06,1.35)^**^
OB	1.16 (1.06,1.62)^**^	1.16 (1.03,1.24)	1.16 (1.03,1.23)^*^	1.12 (1.02,1.24)^**^	1.13 (0.98,1.25)^*^
Pvalue	0.001	<0.001	0.001	<0.001	0.002
LDL-C (mmol/L)					
CON	2.61 (2.08,2.91)	2.57 (1.98,2.91)	2.43 (2.01,2.83)	2.45 (2.05,2.86)	2.36 (2.03,2.75)
OW	2.88 (2.37,3.18)^*^	2.78 (2.32,3.12)	2.70 (2.27,3.09)	2.64 (2.32,3.16)	2.69 (2.36,3.08)^*^
OB	2.98 (2.47,3.21)^*^	2.98 (2.42,3.30)	2.99 (2.38,3.17)^*^	2.81 (2.32,3.15)^*^	2.99 (2.28,3.26)^*^
Pvalue	0.046	0.062	0.038	0.046	0.015
FBG (mmol/L)					
CON	5.09 (4.83,5.57)	4.97 (4.31,7.04)	5.20 (4.66,6.07)	4.66 (4.04,5.21)	4.89 (4.66,5.17)
OW	5.28 (5.02,5.71)	5.67 (4.99,6.73)	5.62 (5.18,6.61)^*^	5.19 (4.59,5.56)^*^	5.24 (4.94,5.61)^*^
OB	5.48 (5.17,5.77)^*^	5.96 (5.13,7.65)^*^	5.60 (5.13,7.18)^*^	5.04 (4.65,5.73)^*^	5.26 (4.93,5.66)^*^
Pvalue	0.016	0.043	0.053	0.022	<0.001
FINS (uIU/mL)					
CON	6.18 (4.25,8.67)	34.34 (27.29,48.51)	30.95 (23.62,54.03)	13.8 (6.15,25.41)	5.97 (4.0,10.46)
OW	10.44 (8.17,12.84)^*^	58.44 (38.69,80.51)^*^	46.16 (34.55,106.70)^*^	17.12 (8.48,40.45)^*^	9.44 (6.39,15.73)^*^
OB	11.52 (7.71,14.32)^**^	61.39 (37.59,87.38)^*^	56.66 (31.10,91.28)^*^	15.92 (10.34,34.93)^*^	10.68 (7.66,18.46)^**^
Pvalue	<0.001	0.005	0.025	0.037	0.001

Medians (interquartile range) for non–normally distributed variables. ^*^P < 0.05 vs. CON group; ^**^P < 0.001 vs. CON group.

CON, control group; OW, overweight group; OB, obese group; FBG, Fasting Blood Glucose; FINS, fasting insulin; TC, total cholesterol; TG, triglyceride; HDL-C, high-density lipoprotein-cholesterol; LDL-C, low-density lipoprotein-cholesterol.

**Table 3:** Correlation analysis between Metrnl and clinical indicators.

Variable	r	*P*value	Variable	r	*P*value
Age	-0.155	0.114	PHTG	-0.473	<0.001
Sex male	0.026	0.795	TC	-0.146	0.138
BMI	-0.265	0.006	TG	-0.370	<0.001
WHR	-0.215	0.028	HDL-C	0.060	0.540
WHtR	-0.255	0.009	LDL-C	-0.175	0.074
FBG	-0.116	0.241	Scr	-0.354	<0.001
FINS	-0.261	0.007	eCCr	0.238	0.015
HOMA-IR	-0.277	0.004	β2-MG	-0.354	<0.001
TyG	-0.359	<0.001	CysC	-0.317	<0.001

BMI, body mass index; WHR, waist-to-hip ratio; WhtR, waist-to-height ratio; FBG, fasting blood glucose; FINS, fasting insulin; HOMA-IR, homeostasis model assessment of insulin resistance;TyG index, triglyceride–glucose index;PHTG, postprandial hypertriglyceridemia; TC, total cholesterol; TG, triglyceride; HDL-C, high-density lipoprotein-cholesterol; LDL-C, low-density lipoprotein-cholesterol; Scr, serum creatinine; eCCr, endogenous creatinine clearance rate; β2-MG,β2-microglobulin; CysC, cystatinC.

**Table 4:** Binary logistic regression analysis of influencing factors for PHTG.

Variable	*B*value	SE	*P*value	OR (95%CI)
Model 1				
Sex male	0.838	0.408	0.040	2.312(1.039-5.145)
Age	0.025	0.018	0.176	1.025(0.989-1.062)
BMI	0.693	0.261	0.008	2.0 (1.198-3.336)
HOMA-IR	0.427	0.162	0.008	1.533(1.116-2.105)
TG×10	0.570	0.104	<0.001	1.769(1.443-2.168)
HDL-C eCCr	-2.345 -0.014	0.851 0.008	0.006 0.079	0.096(0.018-0.508) 0.986(0.970-1.002)
Metrnl	-1.557	0.361	<0.001	0.211(0.104-0.427)
Model 2				
TG×10	0.695	0.155	<0.001	2.005(1.480-2.715)
Metrnl	-1.595	0.580	0.006	0.203(0.065-0.633)

Model 1: Univariate logistic regression analysis. Model 2: Multivariate logistic regression analysis adjusted for sex, age, BMI, HOMA-IR,eCCr and HDL-C. To facilitate the interpretation of the odds ratio (OR), the TG variable was scaled by a factor of 10. The reported odds ratio therefore represents the estimated change in the odds of the outcome per 10-unit increase in the original TG concentration.

BMI, body mass index; HOMA-IR, homeostasis model assessment of insulin resistance; TG,triglyceride; eCCr, endogenous creatinine clearance rate; HDL-C, high-density lipoprotein-cholesterol; Metrnl, meteorin-like protein.

### 3.5 ROC curve and correlation analysis of Metrnl and PHTG

**Figure 4:**
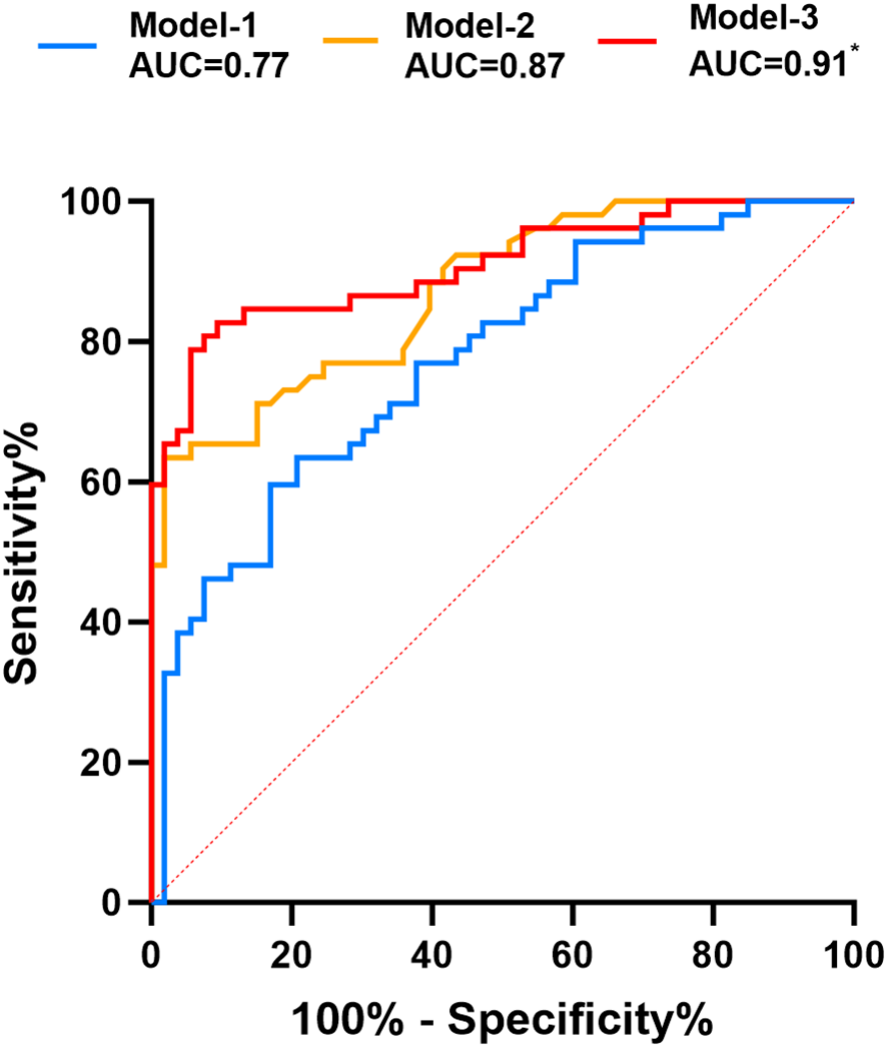
ROC curves of predictive models for PHTG. Comparison of three predictive models for PHTG. Model 1 represents the Metrnl independent prediction, the curve takes a negative value of MetrnL. Model 2 represents the fasting TG independent prediction, and Model 3 represents the combined Metrnl and fasting TG prediction. AUC, Area Under the Curve, is a core index for evaluating the predictive performance of the model, with a larger value indicating better predictive and discriminatory ability of the model. ^*^P=0.001, *vs.* Model-1.

To further quantify the contribution of fasting TG as a risk factor and Metrnl as a protective factor, a predictive model for diagnosing PHTG was established. The independent predictive model for Metrnl was designated as Model-1, the independent predictive model for fasting TG as Model-2, and the combined predictive model of Metrnl and fasting TG as Model 3. ROC curve analysis was performed for all three models. Model 1 had a cutoff value of 2.11 ng/mL, with an AUC of 0.773, sensitivity of 63.5%, and specificity of 79.3%. Model 2 had a cutoff value of 1.21 mmol/L, with an AUC of 0.871, sensitivity of 63.5%, and specificity of 98.1%. Model 3 achieved an AUC of 0.908, which was significantly higher than those of Model 1 and Model 2, with sensitivity increased to 82.7% and specificity to 90.6% (**Table 5**, **Figure 4**). The predictive model integrating Metrnl with fasting triglyceride levels shows improved accuracy in forecasting PHTG.

**Table 5:** ROC curve analysis of models predicting PHTG.

Model	AUC(95%CI)	Cutoff value	Youden’s index	Sensitivity	Specificity	*P* value
Model-1	0.773(0.685-0.861)	2.11(ng/ml)	0.428	63.5%	79.3%	<0.001
Model-2	0.871(0.807-0.936)	1.21(mmol/L)	0.616	63.5%	98.1%	<0.001
Model-3	0.908(0.806-0.965)^*^	—	0.733	82.7%	90.6%	<0.001

Model-1: Metrnl for predicting PHTG; Model-2: Fasting TG for predicting PHTG; Model-3 Combined Metrnl and fasting TG prediction. ^*^P<0.001, vs. Model-1.

## 4 Discussion

At present, fasting serum TG levels are still used as the clinical standard for diagnosing hypertriglyceridemia (HTG). However, because the body remains in a postprandial state for most of the day, postprandial lipid levels are more closely associated with cardiovascular disease and serve as better indicators of average lipid exposure (19–22). In clinical practice, we have observed that overweight and obese individuals frequently present with HTG, and their risk of ASCVD is significantly higher than that of the general population. Nevertheless, overweight and obese individuals with normal fasting lipid profiles are often considered “metabolically healthy obese,” and their ASCVD risk may consequently be underestimated. Therefore, postprandial HTG in overweight and obese populations requires greater attention and further investigation (23). In our team’s previous study using a standardized OFTT, we found that BMI was closely associated with postprandial HTG^3^. Individuals with elevated BMI can thus be considered key groups for monitoring postprandial HTG. At present, studies focusing specifically on postprandial HTG in individuals with high BMI are limited. The latest diagnostic guidelines for overweight and obesity recommend using BMI as the primary classification criterion, supplemented by at least one anthropometric index (e.g. WHR or WHtR) as an auxiliary measure for defining obesity (1). In this study, participants were divided into overweight and obese groups based on BMI cut-off points of 24 kg/m^2^ and 28 kg/m^2^, respectively. WHR and WHtR were measured and calculated within each group as auxiliary diagnostic criteria for overweight and obesity. The results showed that WHR and WHtR in the overweight and obese groups were significantly higher than in the normal-weight group. The mean WHtR in both groups exceeded 0.5, meeting the diagnostic cut-off point for central obesity (24), which was consistent with the BMI-based classification. Therefore, BMI grouping was used as the main criterion for defining overweight and obesity in this study. In 2016, the European expert consensus defined PTG > 2.0 mmol/L at any time after any meal as postprandial HTG (19). A domestic study on non-fasting HTG in overweight individual (3, 25), used two cut-off points (2.0 and 2.26 mmol/L), as recommended by the European Atherosclerosis Society and the American Heart Association, to diagnose postprandial HTG. It was found that even when fasting TG concentrations were within the normal range, most overweight individuals exhibited PTG > 2.0 mmol/L at 4 hours after breakfast. These findings suggested that diagnosing HTG in overweight individuals should rely more on PTG values, and a cut-off point of 2.0 mmol/L is appropriate for defining postprandial HTG. Similarly, the European consensus on postprandial HTG recommended a PTG cut-off of 2.0 mmol/L as the optimal threshold for predicting cardiovascular risk (15). Therefore, in the present study, PTG > 2.0 mmol/L was used as the diagnostic cut-off point for postprandial HTG in overweight and obese individuals.

All participants in this study had fasting lipid profiles within the normal clinical range (14). Fasting lipid levels and lipid changes 1 to 4 hours after a meal were used as assessment criteria for early lipid metabolic disorders. The TyG index was used to evaluate the early metabolic phenotype of obese individuals (26), while HOMA-IR was used to assess the degree of insulin resistance. After BMI-based grouping, fasting TG, INS, HOMA-IR, and the TyG index were significantly higher in the overweight and obese groups compared with the control group, while HDL-C was significantly lower. These findings indicate that early lipid metabolism abnormalities and insulin resistance were already present in overweight and obese individuals. Previous studies have demonstrated that for general ASCVD risk screening, non-fasting blood samples provide prognostic value comparable to that of fasting samples. Given practical considerations and the potential to improve patient compliance, non-fasting sampling is recommended (27). Postprandial triglyceride (PTG) levels rise modestly following a normal meal in healthy individuals. In contrast, overweight and obese individuals demonstrate a markedly greater PTG increase and a delayed clearance phenomenon (28). In this study, a standardized and optimized high-fat meal was used for the OFTT. The results showed that the incidence of PHTG in the overweight and obese groups was 1.59 and 2.03 times higher, respectively, than that in the control group. These findings suggest that in overweight and obese individuals, PHTG should be emphasized more strongly than fasting lipid levels when assessing the risk of ASCVD.

In obesity research, numerous adipokines have been identified as important regulators of lipid metabolism and contributors to the progression of obesity-related complications (29). With the development of genomics and metabolomics, the novel adipokine Metrnl has emerged as a potential key player in metabolic homeostasis. In a study of overweight individuals, circulating Metrnl levels were positively correlated with HDL-C and negatively correlated with LDL-C, small dense LDL, TG, and TC (11). Experimental studies have demonstrated that Metrnl regulates energy metabolism and improves glucose homeostasis in obese mice through multiple pathways (30), enhances pancreatic β-cell function (31), and exerts insulin-sensitizing effects (32, 33). Consistent with these previous findings (6, 34), the present study showed that circulating Metrnl levels in overweight and obese groups exhibited a downward trend, with levels in the obese group significantly lower than those in the control group. Metrnl was also negatively correlated with fasting TG and HOMA-IR, and positively correlated with HDL-C. Monitoring circulating Metrnl levels in overweight and obese individuals revealed a strong association between Metrnl and PHTG. Correlation analyses further demonstrated that fasting Metrnl values were significantly negatively correlated with PHTG, and with postprandial TG, BG, and INS levels, while showing a positive correlation with HDL-C during the OFTT. To further clarify the influencing factors of PHTG in overweight and obese individuals, univariate and multivariate binary logistic regression analyses were conducted. These analyses confirmed that Metrnl acted as a protective factor: for every 1 ng/mL increase in Metrnl, the risk of PHTG decreased by 79.7%.

To further quantify the diagnostic cut-off value for PHTG in overweight and obese individuals, ROC curve analysis showed that the optimal cut-off point of Metrnl as a protective factor was 2.11 ng/mL, with a sensitivity of 63.5% and a specificity of 79.3%. Notably, in overweight and obese individuals with normal fasting lipid profiles, a PHTG prediction model identified fasting TG as a risk factor, with a cut-off value of 1.21 mmol/L. This value is nearly identical to the optimal fasting TG threshold of 1.2 mmol/L recommended in the 2024 European lipid management guidelines (22). These results suggest that maintaining fasting TG levels below 1.2 mmol/L in overweight and obese individuals can substantially reduce the risk of PHTG, with a sensitivity of 63.5% and a specificity of 98.1%. To further enhance diagnostic performance, this study developed a combined prediction model using both fasting Metrnl and TG. The combined model increased sensitivity to 82.7%, which was significantly superior to either marker used independently.

Based on the aforementioned findings, we proceed to discuss the potential mechanisms through which circulating Metrnl participates in regulating lipid and glucose metabolism in overweight and obese individuals, which include the following aspects: Metrnl exerts a pivotal regulatory role in ameliorating lipid metabolic disorders and insulin resistance via conserved signaling pathways and tissue-specific mechanisms (36, 37); at the molecular level, it activates the AMPK-PPARδ pathway to promote fatty acid oxidation in skeletal muscle and suppress lipid-induced inflammation, while also inducing browning of white adipose tissue through the STAT6 signaling axis to enhance energy expenditure, thereby maintaining systemic lipid metabolic homeostasis (34, 38); concurrently, Metrnl contributes to lipid metabolic balance indirectly through its regulation of glucose metabolism—under metabolic stress, it inhibits the transdifferentiation of pancreatic β-cells into α-cells and activates the WNT/β-catenin pathway, which in turn suppresses β-cell apoptosis, promotes proliferation, and ultimately alleviates hyperglycemia-induced β-cell dysfunction (31, 39). Given that the specific mechanisms underlying Metrnl’s involvement in postprandial hypertriglyceridemia (PHTG) remain largely elusive, future studies will involve *in vivo* animal experiments and *in vitro* cellular assays to further elucidate the Metrnl-mediated lipid metabolic pathways in PHTG, thus refining our understanding of its comprehensive biological mechanisms in overweight and obese populations. In conclusion, dysregulation of the adipokine Metrnl in overweight and obese individuals is closely associated with the occurrence of PHTG. Circulating Metrnl may serve as a sensitive and specific biomarker for diagnosing PHTG in this population. From a clinical perspective, enhancing the expression or activity of circulating Metrnl may help interrupt the vicious cycle of lipid metabolism abnormalities in overweight and obese individuals. Elevated Metrnl levels can reduce the occurrence of PHTG by improving insulin sensitivity and decreasing TG synthesis and may therefore represent a promising therapeutic target for obesity and its related complications. Furthermore, obesity is a well-established predictor of chronic kidney disease events and progression to renal failure (28). It can promote adipocyte secretion of pro-inflammatory adipokines, mediate inflammation and insulin resistance, and thereby exacerbate renal damage (35). In the present study, early renal injury markers were also assessed in overweight and obese participants and analyzed in relation to circulating Metrnl. The results revealed a negative correlation between Metrnl levels and Scr, β2-MG, and CysC, and a positive correlation with eCCr. Previous research has indicated that Metrnl can preserve mitochondrial integrity by activating the Sirt3 pathway, thereby mitigating renal lipid accumulation (34). However, the specific role of Metrnl in early renal injury among overweight and obese populations, as well as the underlying mechanisms governing this association, remain to be fully elucidated in future investigations.

## 5 Conclusion

Overweight and obese individuals with normal fasting lipid profiles are at increased risk of postprandial hypertriglyceridemia (PHTG). In this population, the diagnosis of hypertriglyceridemia should be based more on postprandial triglyceride (PTG) levels rather than fasting triglycerides alone. Reduced circulating levels of Metrnl are significantly associated with early disturbances in lipid metabolism and insulin resistance among individuals with obesity.

Elevated circulating Metrnl levels (cut-off: 2.11 ng/mL) may confer protective effects against PHTG, whereas elevated fasting triglyceride levels (cut-off: 1.2 mmol/L) are linked to an increased risk of PHTG. The combination of circulating Metrnl and fasting triglycerides improves diagnostic sensitivity for identifying PHTG, suggesting added value in risk stratification.

Circulating Metrnl is closely associated with renal function impairment in overweight and obese populations and may play a role in the development of obesity-related kidney disease. However, limitations remain, including the absence of globally standardized assays for circulating Metrnl, its current use restricted to research settings without established clinical utility, and the need for large-scale prospective studies to validate the proposed protective cut-off value.

## Data Availability

The original contributions presented in the study are included in the article/**Supplementary Material**, further inquiries can be directed to the corresponding author.
