# Supplementary material for: Metrnl as a predictive biomarker for postprandial hypertriglyceridemia in overweight and obese populations
Source: Front Endocrinol (Lausanne). 2026 Feb 12;17:1729571. doi: 10.3389/fendo.2026.1729571 (PMC12935597; doi:10.3389/fendo.2026.1729571)
Supplement: Supplementary file 1 [file DataSheet1.pdf]

Table1 Basic characteristics of two groups with various PTG concentrations

Group	Total<br>n=105	NPHTG<br>n=53	PHTG<br>n=52	*P*value
Age (Year)	46(37,55)	45(35,55)	50(39,57)	0.181
Sex male (n%)	42(40)	16(30.18)	26(50)##	0.039
BMI	25.79±3.50	25.01±3.50	26.60±3.44#	0.023
CON(n%)	34(32.4)	23(42.6)	11(21.6)	
OW(n%)	39(37.1)	19(35.8)	20(38.5)	0.025
OB(n%)	32(30.5)	11(20.8)*	21(40.4)*	
WHR	0.85±0.06	0.84±0.06	0.87±0.06##	0.007
WHtR	0.52±0.06	0.51±0.06	0.53±0.06	0.060
SBP(mmHg)	122.11±12.59	120±13.22	124.24±11.55	0.100
DBP(mmHg)	78.73±8.09	77.02±7.25	80.48±8.58#	0.028
FBG(mmol/L)	5.31(5.02,5.67)	5.18(4.89,5.56)	5.47(5.06,5.77)	0.109
FINS(uIU/mL)	8.41(4.84,11.70)	8.14(5.02,11.74)	10.92(7.61,14.20)##	0.006
HOMA-IR	2.03(1.08,3.0)	1.87(1.10,2.70)	2.70(1.68,3.38)##	0.003
TC(mmol/L)	4.40(4.07,4.81)	4.21(3.90,4.75)	4.41(4.01,4.77)	0.358
TG(mmol/L)	1.13(0.78,1.40)	0.78(0.60,0.95)	1.29(0.96,1.47) ##	<0.001
HDL-C(mmol/L)	1.28±0.26	1.35±0.26	1.21±0.23##	0.004
LDL-C(mmol/L)	2.73±0.53	2.64±0.54	2.81±0.52	0.097
Scr(μmol/L)	68.81±13.42	62.91±9.39	74.83±14.30##	<0.001
eCCr(ml/min)	105.65<br>(90.82,126.91)	111.60<br>(96.82,126.91)	100.21#<br>(86.33,127.60)	0.046
β2-MG(μg/L)	39.07<br>(28.33,52.54)	33.02<br>(25.73,40.42)	47.95<br>(36.97,60.48)	<0.001
CysC(ng/ml)	40.48<br>(27.84,57.07)	29.16<br>(23.60,37.80)	56.29##<br>(43.60,82.60)	<0.001
Metrnl (ng/ml)	2.23(1.86,2.97)	2.61(2.14,3.28)	2.02 (1.50,2.30) ##	<0.001

Notes: **P* < 0.05, compared with group CON(n%);

#*P* < 0.05, compared with group NPHTG; ##*P* < 0.01, compared with group NPHTG

Comparison of Adipokine Metrnl Levels and Basic Demographic Data Among PTG Subgroups (Table 1)

Abbreviations: PHTG, postprandial hypertriglyceridemia; NPHTG, non-postprandial hypertriglyc eridemia**;** CON, control group; OW, overweight group; OB, obese group; BMI, body mass ind ex; WHR, waist-to-hip ratio; WhtR, waist-to-height ratio ; SBP, systolic blood pressure; DBP,

diastolic blood pressure; FBG, fasting blood glucose; FINS, fasting insulin; HOMA-IR, homeostasis model assessment of insulin resistance; TC, total cholesterol; TG, triglyceride; HDL-C, high-density lipoprotein-cholesterol; LDL-C, low-density lipoprotein-cholesterol; Scr, serum creatinine; eCCr, endogenous creatinine clearance rate; β2-MG,β2-microglobulin; CysC, cystatinC; Metrnl, meteorin-like protein.

All participants underwent an OFTT and demonstrated good tolerance. The cohort included 53 individuals in the NPHTG group and 52 in the PHTG group. No significant difference in age distribution was observed between the groups. The PHTG group had a higher proportion of males, a greater prevalence of obesity as defined by BMI, and a higher waist-to-hip ratio (WHR) compared with the NPHTG group ($P < 0.05$). In addition, the PHTG group exhibited significantly higher diastolic blood pressure (DBP), FINS, HOMA-IR, and TG levels ($P < 0.01$). Serum creatinine (Scr), CysC, and β2-MG levels were also markedly higher in the PHTG group ($P < 0.001$). Conversely, serum Metrnl concentrations were significantly lower in the PHTG group ($P < 0.001$), while HDL-C levels and eCCr values were reduced compared with the NPHTG group ($P < 0.01$).
Stratification analysis based on the oral fat tolerance test (OFTT) revealed that individuals with postprandial hypertriglyceridemia exhibit elevated fasting triglyceride levels and reduced high-density lipoprotein cholesterol (HDL-C) concentrations. These metabolic abnormalities are strongly associated with a significantly increased risk of atherosclerosis. Compared to the normotriglyceridemic (NPHTG) group, the hypertriglyceridemic cohort demonstrated significantly elevated diastolic blood pressure, increased insulin resistance as quantified by the homeostasis model assessment of insulin resistance and a marked reduction in the adipokine Metrnl. Notably, serological analyses demonstrated significantly higher serum creatinine and cystatin C levels in the postprandial hypertriglyceridemia group, accompanied by an reduction in estimated glomerular filtration rate (eGFR) compared to controls, aligning with early pathophysiological features of obesity-related nephropathy. These findings support comorbid mechanisms linking postprandial dyslipidemia with Overweight and Obese Populations
